# Spray-Dried Nanolipid Powders for Pulmonary Drug Delivery: A Comprehensive Mini Review

**DOI:** 10.3390/pharmaceutics16050680

**Published:** 2024-05-17

**Authors:** Mahmoud H. Abu Elella, Arwa Omar Al Khatib, Hisham Al-Obaidi

**Affiliations:** 1 School of Pharmacy, University of Reading, Reading RG6 6UR, UK; m.h.e.abuelella@reading.ac.uk (M.H.A.E.); a.al-khatib@pgr.reading.ac.uk (A.O.A.K.); 2 Faculty of Pharmacy, Al Ahliyya Amman University, Amman 19111, Jordan

**Keywords:** lung diseases, pulmonary drug route, inhalable lipid nanoparticles, spray drying technique, dry powder inhaler

## Abstract

Lung diseases have received great attention in the past years because they contribute approximately one-third of the total global mortality. Pulmonary drug delivery is regarded as one of the most appealing routes to treat lung diseases. It addresses numerous drawbacks linked to traditional dosage forms. It presents notable features, such as, for example, a non-invasive route, localized lung drug delivery, low enzymatic activity, low drug degradation, higher patient compliance, and avoiding first-pass metabolism. Therefore, the pulmonary route is commonly explored for delivering drugs both locally and systemically. Inhalable nanocarrier powders, especially, lipid nanoparticle formulations, including solid-lipid and nanostructured-lipid nanocarriers, are attracting considerable interest in addressing respiratory diseases thanks to their significant advantages, including deep lung deposition, biocompatibility, biodegradability, mucoadhesion, and controlled drug released. Spray drying is a scalable, fast, and commercially viable technique to produce nanolipid powders. This review highlights the ideal criteria for inhalable spray-dried SLN and NLC powders for the pulmonary administration route. Additionally, the most promising inhalation devices, known as dry powder inhalers (DPIs) for the pulmonary delivery of nanolipid powder-based medications, and pulmonary applications of SLN and NLC powders for treating chronic lung conditions, are considered.

## 1. Introduction

**Figure 1:**
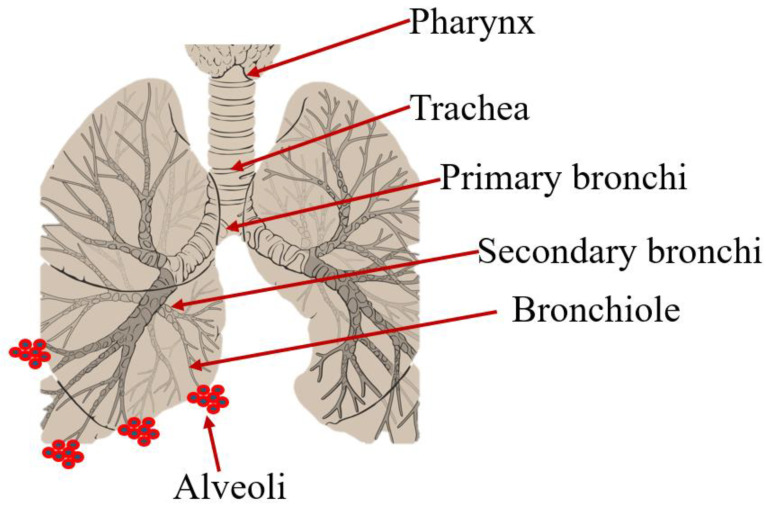
Schematic illustration of the respiratory system.

The lung is an attractive target for drug delivery due to its large surface area, which is about 75–140 m^2^, and a relatively low enzymatic controlled environment for systemic absorption of medications [1]. The lungs, located in the thoracic cavity, consist of major structures, such as bronchi, bronchioles, alveoli, and blood vessels (Figure 1). The airway passages serve to transport air in and out of the lungs while exchanging both O_2_ and CO_2_ with the bloodstream, which is the main responsibility of the alveoli. Additionally, understanding their anatomy is vital in comprehending respiratory functions, diseases, and treatments [2,3].

The lungs have evolved protection mechanisms to prevent the invasion of unwanted airborne particles from invading the body, as they are a major port of entry. Despite their protective mechanisms, the lungs are consistently exposed to a range of stress factors, including chemical, microbial, and physical influences throughout life. Inhalation of various harmful toxins and microorganisms leads to the development of lung diseases. Lung diseases, including lung cancer, asthma, mycobacterium-based tuberculosis, COPD, and cystic fibrosis, are diseases that target the various regions of the lungs, particularly the airways [4,5,6]. Chronic inflammation typically elevates the susceptibility to debilitating lung conditions.

They pose significant public health challenges and burden healthcare systems considerably. Morbidity rates for these conditions have seen a sharp increase. As per the reported list by the WHO regarding the top ten reasons for mortality globally, four of them are attributed to lung diseases: lower respiratory conditions, COPD, lung cancer, and TB. They accounted for approximately one-third (31.35%) of the total global deaths [7]. Additionally, chronic lung diseases in the United Kingdom contribute to 24% of mortality cases [8]. A 2017 Global Burden of Disease Study identified that 545 million people suffer from chronic respiratory conditions globally [9].

COPD and asthma, among all inflammatory diseases, collectively impact millions of individuals globally. Chronic inflammatory ailments result from prolonged inflammatory processes caused by numerous heightened expressed genes associated with inflammation [4]. While a range of pharmacotherapeutic strategies, including antibiotics, peptides, and genetic therapy, such as siRNA and DNA, are utilized for treating lung diseases, these approaches provide relief from symptoms rather than achieving full disease eradication [10]. 

This review focuses on the development of inhalable lipid nanoparticle powders, which are fabricated using a spray dryer for pulmonary administration by using the most promising powder-medication-based inhalation device, known as dry powder inhalers (DPIs). Furthermore, it presents recent progress made in the pulmonary applications of SLN and NLC powders for the treatment of chronic lung diseases.

## 2. Pulmonary Drug Delivery and Use of Nanocarriers

**Figure 2:**
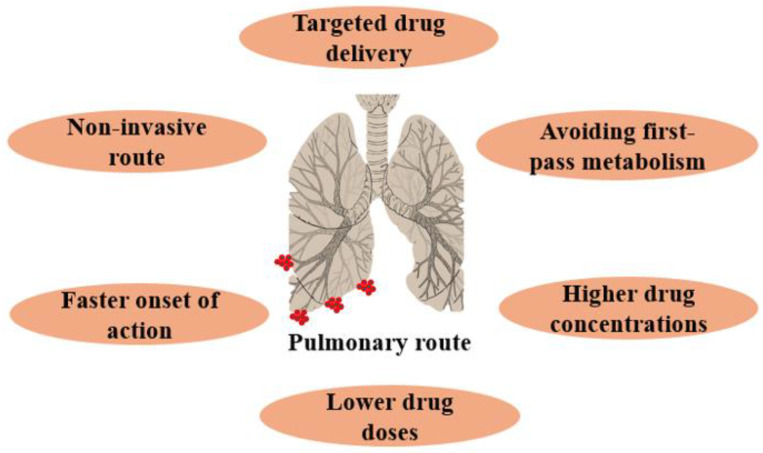
Benefits of the pulmonary drug delivery route.

The pulmonary drug administration route, involving direct inhalation of drugs into the lungs, is highly effective for drug delivery, particularly for treating lung diseases. It rapidly concentrates medication at the target site while minimizing systemic drug levels. Compared to traditional delivery methods, it offers distinct advantages (Figure 2), such as the lungs’ large surface area, a non-invasive route, high epithelial permeability, targeted drug delivery, reduced enzymatic activity, minimal degradation, improved patient compliance, and bypassing first-pass metabolism, resulting in an immediate therapeutic response [11,12]. 

In addition to the extensive surface area of the lung, the alveolar region also benefits from a robust blood supply and a permeable membrane (0.2–0.7 µm), facilitating quick absorption. This strategy guarantees the swift and successful delivery of drugs to their intended site of action [11,13].

As a result, pulmonary delivery is gaining traction not only for treating airway diseases locally but also for administering drugs systemically, particularly for poorly water-soluble medications with limited bioavailability through alternative routes, such as oral administration [14]. 

For the localized management of airway diseases, pulmonary administration distinguishes itself by directly reaching the lung epithelium. This results in a swift onset of action and reduces the required dosage compared to traditional administration methods, like oral delivery [15,16].

To optimize the efficiency of respiratory delivery, it is essential to employ a well-suited drug formulation possessing suitable physicochemical properties. Nanotechnology is attracting considerable interest in addressing respiratory conditions. The development and application of nanocarriers hold great potential for enhancing the human quality of life. Nanomedicine entails the pharmaceutical application of nanotechnology to enhance patient healthcare [17]. Nanocarriers for pulmonary application have garnered significant attention over the past two decades because they must meet several criteria, including biocompatibility and biodegradability, adequate drug capacity, shielding the degradation of the drug, and good aerosolized stability.

Various nanocarriers, including SLNs, SLMs, liposomes, pro liposomes, polymeric NPs, polymeric MPs, and polymeric-coated NPs, have been explored in recent decades. Emerging therapies encompass inhalable nanocarriers designed to target inflammatory receptors at the early stages of infection. This approach holds promise as an ideal strategy for preventing disease progression and tissue damage. Recent research indicates that lipid NPs can meet these requirements, revealing their availability for the pulmonary administration route.

Interest in SLN and NLC as substitutes for other nanocarriers, such as liposomes, nanoemulsions, and polymeric-based nanoparticles, is growing, not only for pulmonary administration but also for other drug administration routes, including ocular, oral, dermal, and parenteral routes [18,19,20,21,22]. This has included, for instance, the approval of PulmoSphere™ technology used in products like Pulmozyme^®^ and TOBI^®^ Podhaler™, which utilize liposomal formulations for pulmonary delivery to treat conditions like cystic fibrosis. These products demonstrate the clinical viability and effectiveness of nanoparticle-based inhalation therapies in enhancing drug delivery to the lungs, thus improving patient compliance and reducing systemic side effects.

Furthermore, the successful application of liposomal amikacin (Arikayce^®^) for the treatment of lung infections associated with cystic fibrosis and non-tuberculous mycobacterial infections further underscores the potential of lipid-based nanoparticles in pulmonary applications. These examples showcase their prospective transformative impact on pulmonary drug delivery systems.

## 3. Lung Surfactant and Impact on Pulmonary Drug Delivery

Lung surfactant is composed of a blend of lipids and proteins situated in the alveolar lining of the lungs. Its function involves reducing surface tension by creating a singular layer at the interface between alveolar air and the liquid. Additionally, lung surfactant plays a crucial role in enhancing the effective dispersion of inhaled drugs delivered to the lung throughout the mucus surface, and it improves the transport of aerosol among different lung regions, leading to more uniform drug distribution across the deeper lung areas [23].

Interestingly, inhaled drugs must initially penetrate the lung surfactant layer to reach the underlying tissue. Therefore, the presence of the surfactant layer affects drug deposition within the lungs in several ways, including diffusion limitation, drug particle size, and extended residence time [24]. In the former, the surfactant layer may serve as a barrier, impeding drugs from accessing the deeper tissues. Consequently, this could result in decreased drug concentrations in the lungs and reduced treatment efficacy. In the case of the drug particle size factor, notably, the size of drug particles influences how deeply they are deposited within the lungs. For example, large particle sizes could get trapped on the surface of the surfactant layer, thus restricting their penetration; conversely, small particles may bypass the layer and reach deeper regions of the lungs. In the latter case, the surfactant layer can extend the duration of drugs within the lungs. This is advantageous for drugs that are quickly expelled, as it enables prolonged exposure to the lung tissue, thereby increasing their effectiveness.

## 4. Inhalation of Nanolipid Powders for Pulmonary Drug Delivery

### 4.1. Lipid Nanoparticles

Lipid-based nanoparticles are vehicles for small molecule delivery, and, in the context of this review, they are used for pulmonary drug delivery. They are spherical-shaped vesicles comprised of ionizable lipids. These lipids, at low pH, carry a positive charge, whereas at physiological pH, they remain neutral, which helps to reduce potential toxic side effects. Due to their small, spherical size, these nanoparticles can deliver drugs into cells via endocytosis and release the cargo into the cytoplasm [25]. Lipid-based drug delivery is a growing area of interest due to its ability to overcome barriers faced by present conventional formulations. They are seen as a suitable replacement for other polymer NPs due to the biocompatible nature of lipids in the body [26].

The initial drug dosage integrating lipid-soluble drugs into droplet-based lipids emerged in the 1960s as the parenteral fat emulsion. Today, this formulation is extensively utilized for the parenteral administration of poorly water-soluble drugs, such as propofol or diazepam [27]. During the 1990s, three research teams, namely, Müller [28], Gasco [29], and Westesen et al. [30], pioneered the first generation of lipid nanoparticles, known as SLNs.

The solid lipid nanoparticle (abbreviated to SLN) category is the first generation of nanolipid carriers. Also referred to as colloidal carriers, SLNs have been developed as an alternative nanocarrier group to preexisting others, such as, for instance, emulsion-, liposome-, and polymer-based NPs. SLNs are essentially lipid-based emulsions in the range of the submicron. The oil is substituted with solid lipids, resulting in a solid lipid matrix at ambient and physiological temperatures [26].

**Figure 3:**
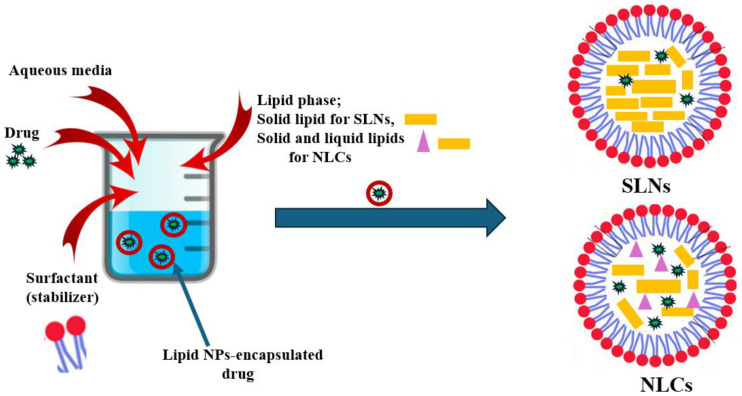
Schematic preparation of two lipid nanoparticle formulations (SLNs and NLCs).

In SLNs (Figure 3), the oil component of the fat emulsion is substituted with a solid lipid or a mixture of solid lipids, resulting in a solid lipid matrix for SLNs at both room and body temperatures. SLNs typically consist of 0.1–30 wt.% dispersed lipid in an aqueous phase composed of surfactant (around 0.5–5 wt.%) as a stabilizer. The average diameter of SLNs typically falls within the range of 40–1000 nanometers [31,32].

SLNs typically offer a combination of advantages, merging the favorable characteristics of fluid-like, lipid-based colloidal particles (such as ingredient biocompatibility) with the straightforward production methods of solid matrix polymeric nanoparticles. This feature facilitates the local and systematic administration of hydrophobic therapeutic agents [33]. In contrast to polymeric nanoparticles, SLNs offer greater safety potential due to several factors: it is a green method without solvent, and it allows for the utilization of biocompatible and biodegradable ingredients (GRAS) [34]. However, there are a few limitations to SLN, such as, for example, its low loaded-drug capacity, due to the change from low lipid modification to a highly ordered, crystallized, firm structure during storage. To address these barriers, a second-generation nanolipid formulation was fabricated [35].

The second generation is called nanostructured lipid carriers (abbreviated as NLCs). These carriers contain a solid lipid matrix at both room and body temperatures, similarly to SLNs, but the difference here is that the matrix comprises a blend of a solid lipid as well as a liquid lipid (Figure 3), such as, for example, oleic acid, miglyol oil, or castor oil. The combination of both lipids creates a low-ordered lipid matrix allowing for high rooming for therapeutic agents. This can be accomplished through different development approaches, such as, for instance, hot or cold high-pressure homogenization, micro-emulsion, emulsification/solvent evaporation, or diffusion techniques [17,36].

**Figure 4:**
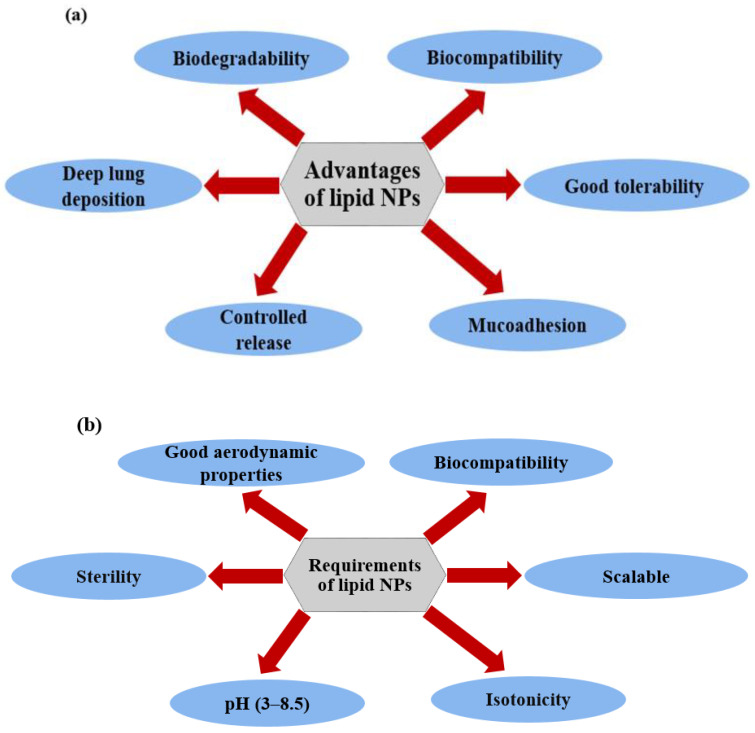
(**a**) Features of SLCs and NLCs for lung drug route, and (**b**) ideal criteria for SLCs and NLCs for pulmonary applications.

For the pulmonary administration route, lipid nanoparticles offer several advantages (Figure 4a). Both SLNs and NLCs are sub-micron-sized, and this feature is beneficial for pulmonary delivery. This property enables nanoparticles to be readily entrapped within particles or aerosolized into droplets, which enables the active compound to be deposited far into the lung. Their size also allows for a longer period of adhesion to the mucosal surface in comparison to larger particles, which allows for longer drug action [17]. This adhesion factor, as well as the accumulation, retention, and prolonged release features of both SLNs and NLCs in the lungs, offer improved and sustained therapeutic outcomes, thereby promoting good patient compliance. It is important to link this back to the chronic indications this could potentially be used for, as many existing treatments require a minimum twice-daily dose thanks to their short lifetime in the lungs [37].

This feature can be particularly significant in curing chronic conditions, as many current inhalation formulations require administration twice daily because of their relatively short duration in the lung. Moreover, because both SLN and NLC are made of biocompatible lipids, this ensures good tolerability in the airways. Additionally, using the biodegradable lipid results in avoiding any toxic degradation products, thereby decreasing the risk of toxic effects and further increasing compliance [38].

### 4.2. Ideal Criteria for Lipid NPs Intended for Pulmonary Applications

To effectively address various lung diseases, the medication needs to primarily target and exert its effects within the lungs. This requirement contrasts conventional approaches to drug and dosage form design, which typically prioritize enhanced absorption and bioavailability throughout the body. However, when a medication designed to cure lung infections is taken up and distributed to other body organs, it can lead to diminished concentrations at the desired location and potentially cause significant adverse effects affecting the gastrointestinal, cardiovascular, and central nervous systems. Hence, for drug administration aimed at targeting lung diseases, both SLNs and NLCs must adhere to specific requirements for ideal pulmonary drug delivery systems, as illustrated in Figure 4b, including biocompatibility, good tolerability, isotonicity, sterility, and a neutral pH value. This is crucial, as the lungs possess limited buffering capacity [39].

To ensure biocompatibility, it is advisable to employ biodegradable and well-tolerated ingredients, including lipids and surfactants. Neutral isotonic carbohydrates are preferable to more highly ionic isotonic salts, like sodium chloride, to mitigate undesired effects. Formulations including an isotonicity around 300 mosmol/kg and a pH range between 3 and 8.5 are desirable [17].

Lipid NPs can be administered in pulmonary applications either as suspensions [40] or converted to inhaled dry powder [41]. These scenarios result in aerosols generated from suspended or dry powder formulations that exhibit a favorable, aerodynamic size to ensure adequate deposition in targeted airway parts. Consequently, the inhaled lipid NP formulations must possess ideal aerodynamic properties, including particle/droplet size and density, mass median aerodynamic diameter (MMAD), and fine particle fraction (FPF). Typically, the aerodynamic aerosol distribution falls between 0.5 µm and 10.0 µm. On the other hand, the ideal aerodynamic size is determined by the intended site of deposition, which varies according to the therapeutic approach. For suspension and powder formulations, the aerodynamic diameters of the inhaled formulations should ideally be below 5 µm to facilitate deep deposition within the lungs [42,43].

Each inhalable formulation requires sterilization utilizing suitable methods, such as sterilized steam, irradiated γ-rays, or a sterilized filtration membrane. Two key considerations must be addressed before the sterilization of lipid NPs. The impact of the sterilization procedure on lipid NPs’ formulation stability, as well as the selection of the optimal sterilization procedure, should consider both the physical and chemical stability of the formulation during the sterilization process [17].

It is not possible to provide a general answer regarding how sterilized steam or gamma-ray irradiation affects the stability of the nanolipid. With γ-ray irradiation, there is a risk of the generation of free radicals from chemically modified drugs due to the formation of free radicals. Additionally, the sterilized filtration membrane is only viable if its size is less than 200 nm. Furthermore, during autoclaving, melting of lipids can occur, followed by recrystallization upon cooling, thus potentially altering the drug release pattern. Additionally, there is a possibility of lipid droplets coalescing before reforming into lipid NPs, resulting in large particles. As a result, autoclaving mainly presents physical–stability challenges, including increased size or aggregation. Cavalli [29], Heiati [44], and Nayak [45] have demonstrated successfully autoclaved SLN and NLC.

### 4.3. Biosafety and Pharmacokinetics of Inhalable Drug-Loaded Lipid Nanoparticle Delivery System

When drugs are provided without proper protection, they are vulnerable to degradation, which can limit their effectiveness. However, utilizing drug carrier systems, such as lipid nanoparticles (LNPs), in inhalation therapy can alleviate these risks. The pulmonary route is a popular method for drug delivery, as it helps to avoid drug loss due to gastrointestinal degradation and first-pass metabolism in the liver. However, the pulmonary tract also contains numerous xenobiotic metabolizing enzymes that are capable of modifying the integrity and pharmacokinetics of inhaled therapeutics. This can be a challenge for drug developers who need to ensure that their therapeutics are deliveredefficiently. Delivering drugs through the pulmonary route requires the drugs to withstand the shear forces experienced during the aerosolization process. Encasing inhaled therapeutics within lipid nanoparticles enhances their protection and helps delay degradation. This also improves the bioavailability of intact compounds and pharmacokinetic profiles. LNPs are a promising drug carrier system for inhalation therapy because they can protect the drug from degradation and modification while also allowing for efficient delivery to the lungs [46,47,48,49].

Moreover, it has been observed that inhalable drug-loaded lipid nanoparticles (LNPs) offer a promising strategy for mitigating the likelihood of inducing an immune response compared to administering drugs without protection. This is because LNPs provide a protective shield for the payload, thereby reducing its recognition as a foreign substance by the immune system. In addition, LNPs decrease phagocytic clearance by alveolar macrophages, leading to a prolonged circulation time and higher drug bioavailability. As a result, patients can benefit from a faster clinical response and require less frequent dosing, thus reducing the risk of severe side effects. This could ultimately contribute to improved patient compliance and better treatment outcomes [50].

## 5. Spray-Dried Nanolipid Powder Formulation for the Pulmonary Route

### 5.1. Spray Drying Technique

Spray drying is the common method used to produce the inhalable powder. The spray-dried inhaled particle’s size significantly impacts local drug deposition in the lungs. Particle sizes smaller than 5 μm have been noted to exhibit a strong correlation with whole-lung deposition. Spray drying is a commonly employed drying approach in pharmaceutical cosmetics and food applications for the development of various controlled particle sizes with good properties. The combination of formulation and spray drying conditions enables the creation of agglomerated powders possessing cohesive characteristics, like flowable particles, reconstitution behavior, bulky density, and mechanical stability [51].

One significant application of spray drying is the formation of lipid nanoparticle powders. Spray drying is a scalable and commercially viable technique to produce nanolipid powders. It can handle large volumes of feed solutions, making it suitable for industrial-scale manufacturing. Nanolipid powders have gained considerable attention due to their potential in various fields, including drug delivery, nutraceuticals, and the encapsulation of bioactive compounds. Moreover, the spray drying process is relatively fast and cost-effective compared to other techniques, such as microfluidics or solvent evaporation methods. It can be used with a wide range of lipid-based materials that are compatible with various excipients, surfactants, and active ingredients, allowing flexibility in the formulation design of nanolipid powders [52,53,54,55].

Spray drying has been extensively used to encapsulate various active ingredients, including drugs and functional lipids. Lipid nanoparticle powders are designed to encapsulate hydrophobic or lipophilic compounds. They are typically composed of lipids and surfactants, which self-assemble to form nanoparticles with a lipid core and a stabilizing shell. They offer numerous advantages, such as biocompatibility, controlled release, protection of encapsulated compounds, and the ability to target specific tissues [56].

**Figure 5:**
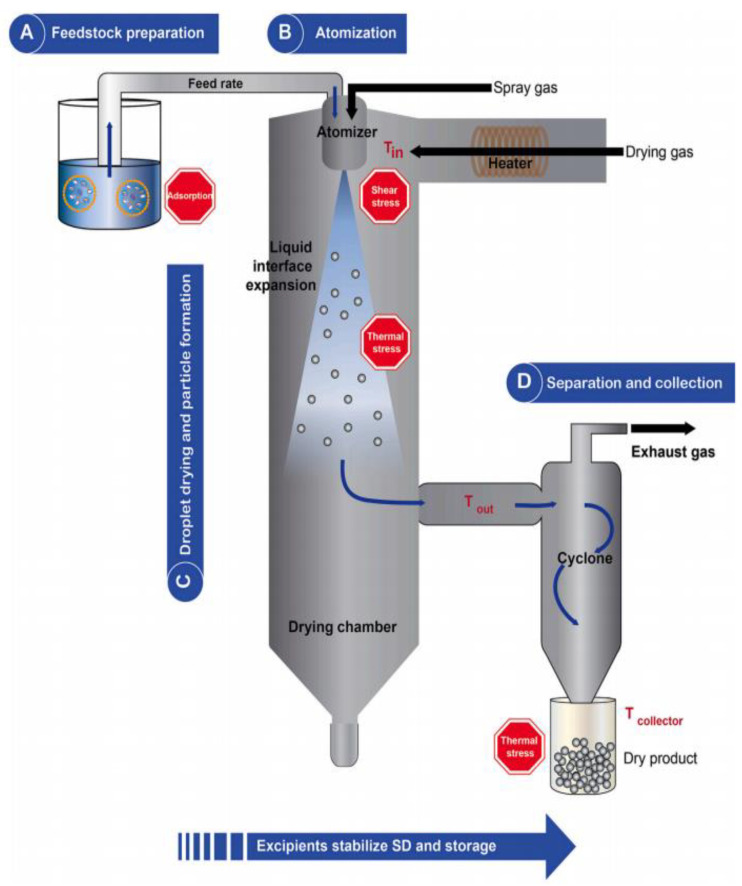
Schematic illustration of spray drying technique. Reproduced with permission from Friis, K.P., Journal of Controlled Release, published by Elsevier, 2023 [57].

The spray drying technique is composed of different parts, including a feed solution, a pump, an atomizer nozzle, a drying chamber, and a cyclone (Figure 5). The spray dryer operates through a series of four stages. First, the liquid forced for drying is atomized into a powder form, followed by the interaction between the hot gas and the nebulized liquid. Then, the solvent evaporates, and, finally, the dried product is separated in the cyclone [54,57]. The process revolves around atomizing a liquid within the drying chamber while hot gas circulates, thus rapidly drying the liquid as the nebulized droplets are exposed to a significant surface area.

The preparation of spray-dried nanolipid powders involves several steps. The lipid components, along with the active ingredient, are dissolved or dispersed in an appropriate solvent to form a feed solution. The selection of lipid components and surfactants is crucial in determining the characteristics and stability of the resulting nanolipid powders. The feed solution is atomized into fine droplets using various techniques, such as pressure nozzles or rotary atomizers [54,58]. The droplet size is a critical parameter that affects the final particle size and encapsulation efficiency. The droplets are exposed to a stream of hot air, leading to the rapid evaporation of the solvent. As the solvent evaporates, the lipid components self-assemble to form nanoparticles. The solidified particles are collected using a cyclone or a filter system. Further processing, such as milling or sieving, may be required to achieve the desired particle size distribution [59]

Spray drying has a significant impact on the properties of nanolipid powders, including particle size, morphology, encapsulation efficiency, and stability. By manipulating the spray drying parameters, like the feed concentration and its flow rate, as well as the drying conditions, the particle size of nanolipid powders can be controlled within the desired range [60]. Smaller particle sizes generally lead to increased surface area and improved bioavailability. The loading capacity of nanolipid powders is based on the selection of lipids and surfactants and the properties of the active ingredient. Proper selection of drying conditions is crucial to maintain the integrity of lipid structures and prevent the degradation of encapsulated compounds. Additionally, spray drying provides a high surface-to-volume ratio, facilitating efficient encapsulation of hydrophobic compounds within the lipid powder that has improved solubility and bioavailability, thus enhancing therapeutic efficacy. In food applications, spray-dried nanolipid powders can be used to encapsulate bioactive compounds, such as vitamins, for improved stability and controlled release, resulting in enhanced functional food products [61].

Furthermore, the spray drying technique helps in maintaining nanolipid powders’ stability within the drying condition. The rapid evaporation of the solvent promotes the self-assembly of lipids into nanoparticles, thus preserving the integrity of the lipid structures. Additionally, the conversion of the liquid feed into solid particles reduces the chances of chemical degradation or physical changes in the encapsulated compounds [53], resulting in enhanced stability. The atomization process generates fine droplets with a large surface area, thus providing efficient encapsulation of active ingredients within the lipid core of the nanolipid powders and resulting in an improvement of the solubility and bioavailability of encapsulated compounds [62].

### 5.2. Inhalation Devices for Delivery of Spray-Dried Nanolipid Powders

**Figure 6:**
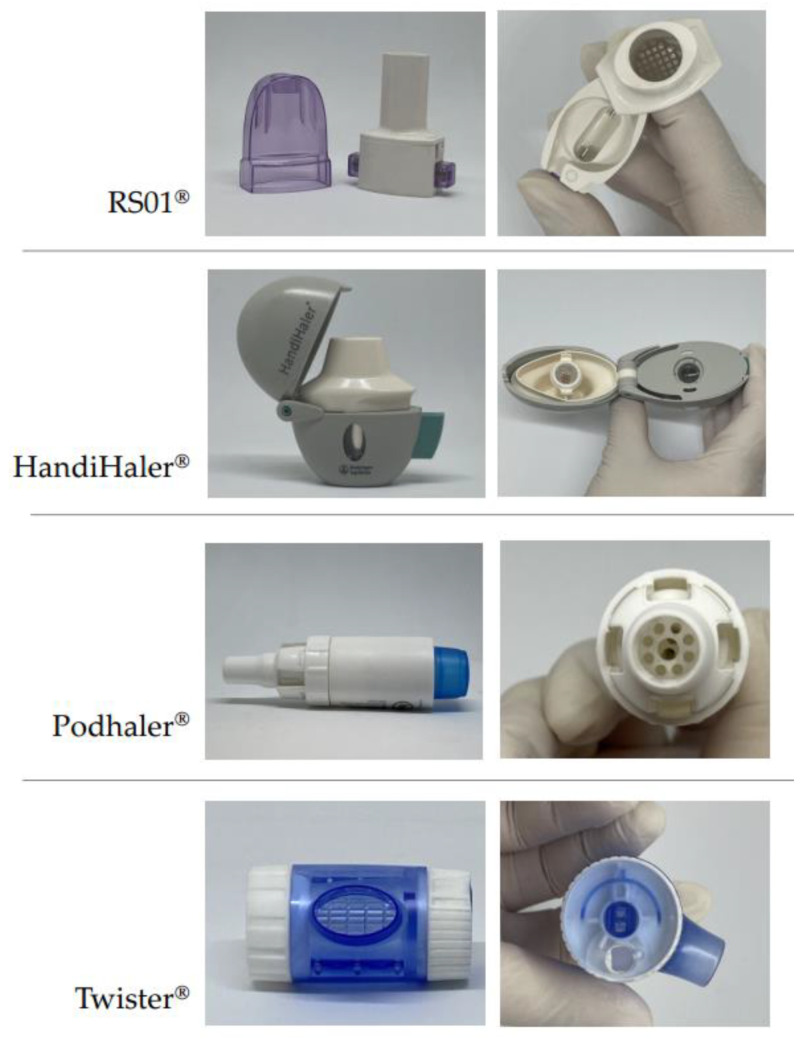
Illustration of various types of capsule-based dry powder inhalers Adapted from MDPI, 2021 [64].

The dry powder inhaler (DPI) is growing in popularity as a method for administering medication to the lungs, providing a convenient and effective option for both local and systemic treatment (Figure 6). These portable devices deliver medication in the form of microscale solid powder, either alone or in the presence of capsules as carriers. DPIs are applied by specially engineered devices that allow patients to inhale drugs directly to their lungs. Conversely, DPI formulations tend to be highly chemically stable compared to liquid formulations, and their development can present significant challenges [63,64].

Their benefits are apparent in the increasing adoption of inhaled treatments for managing obstructive airway conditions in the last decades. While oral bioavailability of fluticasone propionate typically remains below 1%, inhaled forms of fluticasone offer a tenfold increase in bioavailability, with minimal side effects [65]. The effectiveness of a DPI relies on both designed inhalation devices and formulations of powder. DPI designers aim to strike a balance between inhaler resistance and flow rate, as a stronger airflow allows for more consistent and thorough dispersion of the medication, resulting in higher fine particle fractions. However, if the airflow is too rapid, the medication may be deposited in the oropharynx, resulting in lowering the delivered dose of the drug to the lungs [66].

DPI devices exhibit a wide range of designs, and they are categorized into the single-unit type and the multiple-unit type. In the former one, powder is commonly placed into a single-use compartment. This classification can be further divided into three subgroups depending on the technique employed to open the capsule shell and release the powder [64].

Numerous top–down and bottom–up approaches widely utilized to create inhalable powder depend on different costing and compatibility with APIs as well as powder stability. Top–down ones include reducing the particle size to a micro- or nanometer scale, often meeting only basic quality standards, such as jet milling. However, these methods require high energy input, are inefficient, and can present challenges in achieving more demanding performance criteria [67,68]. On the other hand, bottom–up approaches, such as the spray dryer, the spray-freeze dryer, the super critically fluid technique, and the non-wetting template, entail the assembly of the molecular constituent. Consequently, they offer remarkable powder quality, enabling the attainment of more intricate structural designs [68,69].

## 6. Applications of Spray-Dried Nanolipid Powders for Treatment of Lung Diseases via Pulmonary Drug Delivery

Pulmonary drug delivery, as previously mentioned, offers several advantages, making it particularly suitable for delivering drug-loaded nanolipid powders to treat various lung-related diseases. This section outlines the applications of and recent advancements in spray-dried lipid nanoparticle powders (SLNs, and NLCs) for pulmonary drug delivery tailored to treatment-specific lung diseases, such as lung cancer and TB.

### 6.1. Spray-Dried Lipid Nanoparticle Powders for Treatment of Lung Cancer

Lung cancer stands out as a substantial global health issue, holding the position as one of the most common cancers in pharmacokinetics worldwide. The prolonged utilization of traditional anticancer medications has resulted in a notable degree of resistance to lung cancer treatment [70,71]. Consequently, diverse strategies have arisen to combat this resistance, encompassing photothermal methodologies [72], immunotherapy [73], and the simultaneous delivery of chemotherapeutic agents [74]. Nevertheless, the photothermal approach is constrained by several limitations, including the emergence of robust antioxidant systems within cancer cells, challenges in delivering adequate levels of H_2_O_2_, and a low rate of free radical production [72]. Conversely, limitations associated with immunotherapy stem from its adverse effects on the immune system and its restricted efficacy against superficial cancer cells [73]. Intriguingly, the delivery of anticancer drugs amplifies cytotoxicity toward cancer cells with lower dosages, thereby reducing normal toxicity [74].

Tailoring the materials used in SLNs and NLCs can yield significant therapeutic advantages. They have a natural affinity for the lymphatic system, which aids in effectively eliminating hidden cancer cells. In addition, various lipid receptors are often found in abundance on the surface of cancer cells, enabling targeted delivery of anticancer agents [75,76,77]. For example, Jyoti et al. [78] investigated the effectiveness of using spray-dried SLN powders made of stearic acid and egg phosphatidylcholine to release 9-Bromo-noscapine (9-Br-NOS) for lung cancer treatment. The researchers utilized the nanoemulsion technique and incorporated spray-dried lactose to modify the drug release properties. The results showed that the 9-Br-NOS-SLNs had an average particle size of 13.4 nm, an aerodynamic size of 2.3 μm, and a negative charge of −9.54 mV. The study also found that the SLNs had excellent apoptotic effects, were non-toxic, and had efficient cellular uptake in A549 lung cancer cells. Pharmacokinetic and distribution analyses demonstrated a 1.12- and 1.75-fold improvement in the half-life of the drug powder, leading to rapid dispersion, significant dissolution, and deep lung deposition of SLC powders following inhalation administration.

In the same vein, Bakhtiary et al. [79] engaged in the development of a DPI containing SLN powders composed of compritol and poloxamer 407 for the delivery of erlotinib to treat lung cancer. The nanolipid powder formulation achieved a high drug encapsulation efficacy of approximately 78.21%, resulting in enhanced cytotoxicity. This was confirmed by using the MTT method on A549 cells. The powders had an optimal flow (FPF of 30.98%) and an aerodynamic size of about 3.9 μm with the use of mannitol, allowing for deep lung inhalation and effective cancer treatment.

In a related study, Kaur et al. investigated the use of spray-dried NLC powders for delivering paclitaxel (PTX) via inhalation to combat drug-resistant lung cancer. The powders were formulated with glyceryl monostearate, oleic acid, and mannitol to enhance the flow properties, resulting in powders with the ideal aerodynamic size of 3.5 μm. In vitro analysis showed drug release levels of 64.9%, 62.3%, and 59.7% for NLCs loaded with Tween 80, Tween 20, and Tween 60, respectively, over 72 h. In vivo experiments using a DPI device on Wistar rats revealed significant improvement in drug localization within the lungs, effectively treating drug-resistant lung cancer by inhibiting P-glycoprotein inhibitors’ efflux through the use of surfactant-based pulmonary delivery systems [80].

In a concurrent study, Nafee and colleagues incorporated myricetin into spray-dried SLN-based phospholipid Lipoid-S100. The resulting powders, which measured 2.7 μm, exhibited excellent flow characteristics, with a fine particle fraction (FPF) of approximately 81.23%. Additionally, more than 80% of the drug was released within 8 h, and the encapsulation efficiency was approximately 93%, indicating a deep deposition in the bronchial region. It is noteworthy that cytotoxicity was increased, possibly due to enhanced cellular uptake, which was confirmed through confocal imaging and dual fluorescence sensing and resulted in a doubled fluorescence signal [81].

In a different study focused on researching the aerosolization of co-delivered PTX and DOX for treating lung cancer by using inhalable spray-dried NLC powders made of soya lecithin and oleic acid, by employing the cremophor EL surfactant, the research showed an improvement in the drugs’ antitumor efficacy. Furthermore, an in vivo drug distribution analysis of inhalable NLCs/DPI on Wistar rats demonstrated increased drug distribution within the lungs compared to administering the drugs alone [82].

Recently, the pioneering work of Satari N. and his co-workers [83] has investigated diverse pulmonary delivery systems based on inhalable nanolipid powders for anticancer drugs for lung cancer therapy. They have reported the development of a DPI formulation containing gefitinib-loaded stearic acid SLNs targeted with glucosamine to deliver anticancer-agent-like gefitinib directly to lung tumors. The findings displayed enhanced anticancer efficacy compared to free gefitinib, with the enhanced uptake of targeted SLNs in A549 cells compared to non-targeted SLNs. Additionally, the developed mannitol-based microparticles exhibited favorable aerodynamic characteristics, with an acceptable MMAD of 4.48 μm and an FPF of 44.41%.

### 6.2. Spray-Dried Lipid Nanoparticle Powders for Treatment of Tuberculosis

Tuberculosis is considered a significant global public health challenge despite the significant progress achieved in the field. It continues to be one of the leading lung-infectious causes of death and illness worldwide, claiming the lives of nearly 2 million individuals annually. Mycobacterium tuberculosis is the pathogen responsible for causing tuberculosis, an infectious disease. Commonly prescribed drugs for treating tuberculosis consist of rifampicin, pyrazinamide, isoniazid, and ethambutol [84,85].

Traditional methods of drug delivery encompass oral and intravenous administration; nevertheless, these approaches may encounter dose restrictions and undergo first-pass effects in the liver, leading to inadequate drug levels in the lungs. Consequently, this can lead to the emergence of drug resistance and treatment inefficacy. Extended treatment durations and invasive procedures often result in poor patient adherence to the treatment regimen [86,87]. Therefore, pulmonary inhalation for drug delivery presents a promising avenue to tackle these challenges owing to its effective treatment and diagnosis of lung infections. It offers a safe, convenient, and non-invasive diagnostic method [88]. Recently, lipid nanoparticle powders have garnered great attention for delivering anti-TB drugs to the lung via the pulmonary route because of the remarkable above-mentioned properties.

As an example, Gaspar D. and his team fabricated SLN powders composed of glyceryl dibehenate and glyceryl tristearate as a hybrid platform antibiotic drug carrier for the treatment of TB disease via the pulmonary route [89]. The microencapsulated rifabutin (RFB)-loaded SLN powders used FDA-approved excipients, such as mannitol and trehalose, via the spray drying method. An in vivo biodistribution study of inhalable DPI of RFB-loaded powders using BALB/c mice revealed that the antibiotic reached the tested organs via pulmonary administration after 15 and 30 min. Furthermore, the antimycobacterial activity was assessed in a murine infected with TB strain H37Rv, showing improved efficacy against TB infection compared to healthy animals.

In the same context, Nemati et al. [90] explored the pulmonary drug administration route for TB therapy using a DPI containing ethambutol drug-loaded Compritol SLN powders prepared via a hot homogenization/ultrasonication-assisted spray drying technique. Through MTT assay, the DPI formulations demonstrated excellent compatibility and non-toxicity. The resulting spray-dried powders exhibited notable flowability and inhalable characteristics, with an MMAD value of 4.148 μm and an FPF of 30.9%. Table 1 shows the different spray-dried nanolipid powders for pulmonary drug delivery via DPI for lung cancer and TB treatment.

## 7. Conclusions and Future Prospectives

Lipid nanoparticle powders stand out from other nanocarriers due to their numerous advantages, including excellent tolerability, as they consist of biodegradable ingredients, biocompatibility, facile production without the need for organic solvents, cost-effectiveness, good mucoadhesion, stability, and controlled release properties.

Moreover, this review pointed to the specific requirements of SLN and NLC powders for an ideal pulmonary drug delivery system aimed at targeting lung diseases. For instance, these include biocompatibility, good tolerability, isotonicity, sterility, a neutral pH value, adequate drug loading, protection of the drug from degradation, stability during aerosolization, and good aerodynamic properties.

This review also listed the advantages of the DPI-based spray dryer technique for the delivery of lipid nanoparticle powders through the pulmonary route, such as its portable design, the breath-activated mechanism that eliminates the need for patient hand–mouth coordination, and no propellant necessary.

The preceding studies discussed in this review showcase that pulmonary drug delivery enables the administration of various chemotherapy agents and anti-tuberculosis drugs via spray-dried nanolipid powders, effectively addressing both lung cancer and tuberculosis (TB), respectively. Furthermore, numerous studies have demonstrated the low toxicological potential of lipid nanoparticle powders administered via the pulmonary route.

The field of pulmonary drug delivery is constantly evolving, with new developments and advancements in technology. However, to enhance the effectiveness of such delivery systems, it is imperative to consider the long-term effects of repeated pulmonary administration of lipid nanoparticles. Currently, there is a lack of research in this area, and future studies should prioritize identifying biocompatible excipients to enhance the stability, absorption, and aerosol performance of inhaled biologics. Moreover, particle size and the presence of excipients play a crucial role in enhancing the therapeutic effects and achieving superior targeting of the drug. Therefore, it is essential to consider these parameters while designing inhalation devices for effective drug delivery. While dry powders as inhalable nanoparticles present unique advantages for targeted drug delivery, they also pose potential challenges, particularly in developing device-based dosage forms for pulmonary drug delivery. Currently, there is no perfect device that can undertake pulmonary drug delivery with ease. Hence, it is crucial to design pulmonary drug delivery devices that are small and simple to ensure patient compliance and ease of use. It should be noted that despite the potential benefits of these devices, their absorption, distribution, and protective capabilities are still in the research phase. Therefore, additional safety testing is required before their clinical application. The potential risk of regional toxicity with prolonged usage remains unknown, emphasizing the need for further research in this area. Overall, the pairing of nanotechnology with the design of inhalation devices holds promising potential for the advancement of pulmonary drug delivery systems.

## Figures and Tables

**Table 1:** Different DPI formulations based on spray-dried nanolipid powders targeting both lung cancer and tuberculosis (TB) treatment via the pulmonary route.

Lipid Formulation	Lipid Composition	Active Ingredients	Lung Disease	Spray Drying Conditions	References
SLN	Stearic acid	9-Bromo-noscapine	Lung cancer	- Spray-dried lactose carrier	[78]
NLC	Glyceryl monostearate and oleic acid	Paclitaxel (PTX)	Lung cancer	- Mannose and leucine as carriers - Inlet temp. is 80 °C - Outlet temp. is 40 °C	[80]
NLC	Soya lecithin and oleic acid	PTX and doxorubicin (DOX)	Lung cancer	- Lactose and leucine (carrier) - Inlet temp. is 80 °C - Outlet temp. is 40–55 °C - Flow rate of 830 L/h	[82]
SLN	Compritol	Erlotinib	Lung cancer	- Mannitol (carrier) - Inlet temp. is 110 °C - Outlet temp. is 65 °C - Feed rate of 10 mL/min - Aspiration rate of 70%	[79]
SLN	Stearic acid and PEG/glucosamine	Gefitinib	Lung cancer	- Mannitol and lactose (carriers) - Inlet temp. is 160 °C - Outlet temp. is 65 °C - Feed rate of 1.5 mL/min - Aspiration rate of 90%	[83]
SLN	Compritol	Ethambutol	Tuberculosis	- Mannitol (carrier) - Inlet temp. is 110 °C - Outlet is 65 °C - Feed rate of 10 mL/min - Aspiration rate of 70%	[90]
SLN	Glyceryl dibehenate and glyceryl tristearate	Rifabutin	Tuberculosis	- Mannitol and trehalose (carriers) - Inlet temp. is 103 °C - Feed rate of 8.5 mL/min - Aspiration rate of 100%	[86]

## Data Availability

Not applicable.
